# Predictors of musculoskeletal disorders among special education teachers in Sabah, Malaysia

**DOI:** 10.1016/j.heliyon.2024.e30873

**Published:** 2024-05-14

**Authors:** Mohammad Saffree Jeffree, Ahmad Asyraf Abdul Rahim, Dayang Maryama Ag Daud, Nicholas Pang, Mohd Fazeli Sazali, Suhaini Sudi, Shye Nee Liau, Eileen Ei Ling Wong, Hanif Fikri Peter, Siti Zuraina Ain Amat, Stephanie Chok, Mohsen M.A. Abdelhafez, Khamisah Awang Lukman, Ismail Saad, Mohd Rohaizat Hassan, Raman Noordin

**Affiliations:** a Department of Public Health Medicine, Faculty of Medicine and Health Sciences, University Malaysia Sabah, 88400, Kota Kinabalu, Sabah, Malaysia; b HEAL Research Unit, Faculty of Medicine and Health Sciences, University Malaysia Sabah, 88400, Kota Kinabalu, Sabah, Malaysia; c Faculty of Science and Natural Resources, University Malaysia Sabah, 88400, Kota Kinabalu, Sabah, Malaysia; d Development Division, Sabah State Health Department, 88590, Kota Kinabalu, Sabah, Malaysia; e Department of Obstetrics and Gynaecology, Faculty of Medicine and Health Science, University Malaysia Sabah, 88400, Kota Kinabalu, Sabah, Malaysia; f Faculty of Engineering, University Malaysia Sabah, 88400, Kota Kinabalu, Sabah, Malaysia; g Department of Public Health Medicine, Faculty of Medicine, Universiti Kebangsaan Malaysia, 56000, Kuala Lumpur, Malaysia; h Faculty of Business, Economics and Accountancy, University Malaysia Sabah, 88400, Kota Kinabalu, Sabah, Malaysia

**Keywords:** Musculoskeletal disorders, Special education teachers, Ergonomics, Psychology, Fitness

## Abstract

Special education teachers encounter considerable occupational challenges, yet there is limited information concerning musculoskeletal disorders (MSD) within this group. Therefore, this study aimed to address this gap by determining the prevalence of MSD, investigating associated factors of MSD, and identifying predictors of MSD among special education teachers. A cross-sectional study was conducted among special education teachers in Kota Kinabalu and Penampang, Sabah. Data were collected through self-administered questionnaires and musculoskeletal fitness assessments. Chi-square tests and independent t-tests were utilized to determine factors associated with MSD, while multiple logistic regression was performed to develop a comprehensive predictive model for MSD, which was then validated and tested for model fitness. A total of 122 individuals participated in the study, yielding a response rate of 95 %. The findings revealed a high prevalence of MSD (77.9 %) among special education teachers, with the lower back, shoulder, neck, knee, upper back, and foot being the most affected regions. The multivariable regression model identified several predictors of MSD, including marital status (adjusted odds ratio [aOR] = 4.78, 95 % confidence interval [CI] = 1.49–15.40), body fat percentage (aOR = 1.06, 95 % CI = 1.00–1.12), teaching in prolonged standing for few days a week (aOR = 3.20, 95 % CI = 0.99–10.29) or every day (aOR = 6.20, 95 % CI = 1.44–26.70), mindfulness (aOR = 0.47, 95 % CI = 0.22–0.98), and back extensor strength (aOR = 5.86, 95 % CI = 1.92–17.92). This study highlights the necessity of implementing interventions focusing on the ergonomic, psychological, and musculoskeletal fitness components to mitigate the prevalence of MSD and improve the overall well-being of special education teachers.

## 1 Introduction

Musculoskeletal disorders (MSD) pose a significant occupational challenge within the teaching profession, particularly among special education teachers who experience a high prevalence of MSD [1,2]. These disorders not only impact teachers' well-being but also significantly burden educational institutions, leading to substantial financial expenses due to sick pay, decreased productivity, retraining, legal fees, and injury benefits [3]. MSD occurs when the body's muscles, ligaments, and tendons are strained during tasks, often involving awkward positions or repetitive motions, leading to discomfort and disability over time. Picture the dedicated teacher tirelessly instructing students while enduring low back pain, epicondylitis, sciatica, carpal tunnel syndrome, or a rotator cuff injury resulting from the physical demands placed on their bodies [4].

The symptoms of MSD vary in intensity and duration, ranging from mild and intermittent to severe, chronic, and debilitating conditions. These symptoms manifest as pain, swelling, numbness, tingling sensations, and limited joint movement in the affected areas [5]. Recent Global Burden of Disease 2019 (GBD) data reveals that approximately 1.71 billion individuals worldwide suffer from MSD, making them the leading cause of Years Lived with Disability (YLD), accounting for 17 % of all YLD in 2019 [6]. The sheer magnitude of these statistics underscores the urgent need to address the impact of MSD on the teaching profession, particularly among special education teachers.

Special education teachers face an additional set of challenges due to the unique nature of their work. In their noble efforts to cater to students with diverse physical and mental needs, these educators often engage in nursing care activities [7]. This includes providing essential support to students who struggle to manage their daily requirements alongside the regular curriculum. From lifting and carrying students to assisting with positioning, transferring between locations, changing diapers, and aiding with feeding, special education teachers go above and beyond to ensure the well-being and progress of their students [2,8]. These tasks expose teachers to various ergonomic risk factors, such as awkward postures, sustaining static positions, and exerting force. The cumulative effects of these demanding tasks contribute significantly to the occurrence of MSD in special education teachers [9].

In Malaysia, special education programs cater to students with various special needs, including visual impairments, hearing problems, intellectual disabilities, learning disorders, autism, and other neurodevelopmental disorders. The Ministry of Education's Special Education Division offers opportunities and facilities through Special Education Schools and Integrated Special Education Programs (ISEP) within primary and secondary regular schools to address these special educational needs [10].

Despite the widespread prevalence of MSD among teachers, limited research has specifically focused on the occurrence of MSD among special education teachers. Additionally, previous studies have predominantly examined individual and ergonomic factors as predictors of MSD, leaving a gap in understanding the role of psychological factors and musculoskeletal fitness in the development of MSD among special education teachers.

To address the knowledge gap, our study aims to determine the prevalence of MSD and explore its associations with sociodemographic factors, ergonomic risk factors, psychological factors, and musculoskeletal fitness factors among special education teachers. By doing so, we aim to develop a comprehensive predictive model for MSD within this specific population. Our findings will not only shed light on the prevalence of MSD but also provide opportunities for targeted programs and preventive approaches to mitigate MSD risk. Ultimately, our study seeks to improve special education teachers' overall health, productivity, and work quality, paving the way for a more supportive and sustainable teaching environment.

## 2 Materials and methods

### 2.1 Study design and participants

A cross-sectional study design was conducted to investigate MSD among special education teachers in primary and secondary schools with the Integrated Special Education Program (ISEP) in Kota Kinabalu and Penampang District, Sabah. The ISEP is a specialized program catering to students with visual impairments, hearing impairments, and learning difficulties [10].

### 2.2 Sample size determination and sampling procedure

Sample size estimation was conducted using the single population proportion formula [11], taking into account a 72 % prevalence of MSD symptoms based on a previous study among special education teachers in Kelantan, a 5 % margin of error, and a 95 % confidence interval (CI) [2]. Initially, a sample size of 122 participants was determined. Then, to account for a 10 % non-response rate, a total of 136 special education teachers were selected using a stratified random sampling method [12].

The sampling process involved school stratification, followed by the random selection of teachers from each school. However, due to non-participation from a few schools and the exclusion of six teachers for various reasons, the final sample size consisted of 122 participants. To be included in the study, teachers had to meet specific eligibility criteria, including being permanent teachers with at least six months of experience in the Integrated Special Education Program. Meanwhile, exclusion criteria encompassed having MSD or injuries unrelated to the workplace within the past six months and being pregnant [8]. Excluding participants with recent non-work-related MSD or injuries helps focus the study on occupational MSD, reducing confounding factors and clarifying the cause of symptoms. Only participants who have provided written consent by signing the consent form were included, respecting their voluntary participation and privacy.

### 2.3 Study instruments

The study utilized two main instruments: self-administered questionnaires and musculoskeletal fitness assessments. The self-administered questionnaires comprised five sections, namely sociodemographic information, MSD assessment, ergonomics risk factors, psychological stress, and mindfulness. These questionnaires were developed based on a comprehensive literature review and received input from field experts. They were validated and showed good reliability and validity [[13], [14], [15]]. A pre-test was conducted to ensure the clarity and appropriateness of the questions. Cognitive interviewing techniques were employed during the pre-test, with teachers filling out the questionnaire and participating in a debriefing session to discuss their responses. The test-retest method with a two-week interval was utilized to assess questionnaire reliability, resulting in acceptable correlation coefficients ranging from 0.75 to 0.85.

The MSD questionnaire was derived from the Cornell Musculoskeletal Discomfort Questionnaire (CMDQ) developed by Dr. Alan Hedge [16]. It has been recognized as a reliable and valid tool for assessing musculoskeletal pain in Malaysian studies, exhibiting high consistency in its sub-scale items (Cronbach's α > 0.95) and favorable Kappa coefficients for frequency, severity and interference scores (ICC = 0.690–0.949, ICC = 0.801–0.979, and ICC = 0.778–0.944, respectively) [13]. Symptoms in 12 body regions were categorized to determine the presence of MSD symptoms in any body part, with the overall MSD serving as the dependent variable for analyzing associated factors and predictors [17].

Ergonomics risk factors were assessed using a questionnaire adapted from the Dutch Musculoskeletal Questionnaire (DMQ) [18]. This questionnaire evaluated the frequency of ten common job tasks performed by special education teachers, specifically targeting main ergonomic risk factors such as prolonged standing and sitting, awkward postures, office strain, and forceful exertion [19]. The frequency of performing these tasks over seven days was categorized as never, a few days a week, and every day [18].

Psychological stress at work was measured using a self-administered questionnaire adapted from the Perceived Stress Scale (PSS-10) [20]. The PSS-10 consists of ten questions assessing the unpredictability, manageability, and burden experienced by special education teachers. In a reliability study among Malaysian nurses, the first component (six questions) exhibited a Cronbach's α coefficient of 0.82, while the second component (four items) had a coefficient of 0.72, indicating satisfactory reliability and validity [14]. The PSS-10 employs a 5-point Likert scale, with higher scores indicating higher levels of perceived stress. Four positively stated statements are scored reversely [20].

The mindfulness questionnaire, adapted from the Mindful Attention Awareness Scale (MAAS) developed by Brown and Ryan, assessed attention and awareness in daily life [21]. The MAAS has demonstrated strong psychometric qualities, including good internal consistency reliability (Cronbach's α = 0.851) [15]. Therefore, it is considered a valuable tool for examining the relationship between mindfulness and perceived stress among teachers [22]. The 15-item scale measured the frequency of attentive states using general and situation-specific statements. Scoring involved calculating the mean performance across the 15 items, resulting in scores ranging from 15 to 90, where higher scores indicated greater mindfulness [21].

Musculoskeletal fitness assessments encompassed evaluations of body composition, strength, endurance, flexibility, and functional movement components. The assessments included body fat percentage, back region fitness (Back Extensor), lateral core fitness (Left and Right-Side Bridge), body core fitness (Plank on Elbow), shoulder flexibility (Left and Right Apley Scratch), and lower limb fitness (Chair Rise in 30 Seconds, Left and Right Single Leg Squat, and Left and Right Forward Lunge) [23,24]. Trained fitness trainers from the Health Through Exercise and Active Living (HEAL) Research Unit at Universiti Malaysia Sabah conducted the assessments at the school premises. Teachers were advised to wear appropriate sports attire, have a meal, and stay hydrated before the tests. Calibrated equipment was used for the assessments, and a comprehensive briefing was provided regarding proper test execution and associated risks. A 5-min warm-up session, including brisk aerobic exercise, was performed to raise heart rate and loosen muscles. Body fat percentage was determined using bioelectrical impedance analysis (BIA), a safe, observer-independent, cost-effective, and convenient method. The Huawei AH100, a single-frequency bioelectrical impedance body composition analyzer, was employed for the measurements [25,26].

### 2.4 Data analysis

The data collected for the study were analyzed using SPSS version 26. Descriptive statistics were utilized to summarize the prevalence of MSD among special education teachers, presenting frequencies and percentages for categorical variables and means and standard deviations for continuous variables. Chi-square tests were employed to examine associations between categorical variables, while independent t-tests were used to explore associations between continuous variables. A two-tailed significance level of p < 0.05 was applied for all analyses. The results were presented using appropriate tables, figures, and narrative descriptions.

Variables demonstrating a significant level below 0.25 in univariable analysis were selected for multiple logistic regression analysis to identify predictors of MSD among special education teachers. The logistic regression analysis employed the Forward and Backward Likelihood Ratio methods. In addition, the models underwent assessment for interaction, collinearity diagnostics, Hosmer-Lemeshow goodness-of-fit, sensitivity, specificity, Positive Predictive Value (PPV), Negative Predictive Value (NPV), F1 Score, classification table, Area Under the Receiver Operating Characteristic (ROC) Curve (AUC) and Cook's influential statistic. Factors with p < 0.05 were considered statistically significant in the final model, and adjusted odds ratios (AOR) with 95 % confidence intervals were utilized to determine the strength of the association.

## 3 Results

### 3.1 Sociodemographic characteristics

The study included 128 special education teachers from 16 primary and secondary schools who met the inclusion and exclusion criteria. Out of these, 122 teachers completed the study, resulting in a response rate of 95 %. Regarding the sociodemographic characteristics of the participants (Table 1), The mean age of the teachers was 38.23 (±7.99) years. The majority of special education teachers were female (73 %) and married (76.2 %). Most teachers had a degree-level education (88.5 %), and a significant portion of them were overweight or obese based on body mass index (BMI) (55.7 %). Most teachers were categorized as non-smokers (91 %), and a relatively large portion of the special education teachers (82 %) had more than five years of teaching experience. For the type of school category, 62.3 % of the special education teachers came from primary school, while the rest were from secondary school (37.7 %). Furthermore, most of the special education teachers had no premorbid (86.9 %).

**Table 1:** Sociodemographic characteristics of respondents.

Variables	Special Education Teachers (N = 122), n (%)
**Age,** mean (SD)	38.23 (7.99)
**Gender**	
Male	33 (27.0)
Female	89 (73.0)
**Marital Status**	
Unmarried	29 (23.8)
Married	93 (76.2)
**Education Level**	
Degree	108 (88.5)
Master or higher	14 (11.5)
**Body Mass Index**	
<25 kg/m^2^	54 (44.3)
≥25 kg/m^2^	68 (55.7)
**Smoking Status**	
Non-smoker	111 (91.0)
Smoker	11 (9.0)
**Teaching Experience**	
<5 years	22 (18.0)
≥5 years	100 (82.0)
**Type of School**	
Primary School	76 (62.3)
Secondary School	46 (37.7)
**Premorbid**	
Yes	16 (13.1)
No	106 (86.9)

### 3.2 The prevalence of MSD

**Fig. 1:**
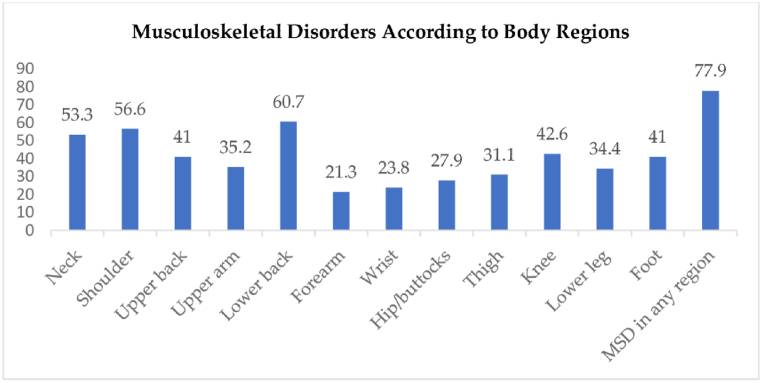
Distribution of MSD according to body regions.

The prevalence of MSD in any body region among special education teachers was 77.9 %. The most commonly affected body regions were the lower back (60.7 %), shoulder (56.6 %), neck (53.3 %), knee (42.6 %), upper back (41 %), and foot (41 %). Other body regions had lower prevalence rates, below 40 % (Fig. 1).

### 3.3 Relationship between associated factors and MSD

Univariable analysis revealed that married teachers (p = 0.019) and those with a BMI of 25 or more (p = 0.008) had a significantly higher occurrence of MSD (Table 2). Teaching in a standing position for more than 2 h daily or a few days a week was also significantly associated with a higher occurrence of MSD (p = 0.046) (Table 3). Psychological factors were found to be significantly associated with MSD. Higher perceived stress levels were associated with a higher occurrence of MSD (p = 0.017), while higher mindfulness levels were associated with a lower occurrence of MSD (p = 0.04) (Table 4). Musculoskeletal fitness factors were also significant predictors of MSD. Higher body fat percentage (p = 0.005) and poor performance in tests assessing back extensor (p = 0.017), left-side bridge (p = 0.040), right-side bridge (p = 0.031), plank on an elbow (p = 0.041), and right Apley scratch (p = 0.030) were associated with a higher occurrence of MSD (Table 5).

**Table 2:** Univariable analysis of the association of sociodemographic factors and MSD.

Item	Musculoskeletal Disorders (N = 122), n (%)		*P*-value^a^
	Yes	No	
**Age,** mean (SD)	38.66 (8.22)	36.70 (7.07)	0.263^b^
**Gender**^c^			0.186
Male	23 (69.7)	10 (30.3)	
Female	72 (80.9)	17 (19.1)	
**Marital Status**^c^			0.019
Unmarried	18 (62.1)	11 (37.9)	
Married	77 (82.8)	16 (17.2)	
**Education Level**			0.452
Degree	83 (76.9)	25 (23.1)	
Master or higher	12 (85.7)	2 (14.3)	
**Body Mass Index**^c^			0.008
<25 kg/m^2^	36 (66.7)	18 (33.3)	
≥25 kg/m^2^	59 (86.8)	9 (13.2)	
**Smoking Status**^c^			0.233
Non-smoker	88 (79.3)	23 (20.7)	
Smoker	7 (63.6)	4 (36.4)	
**Teaching Experience**			0.521
<5 years	16 (72.7)	6 (27.3)	
≥5 years	79 (79.0)	21 (21.0)	
**Type of School**^c^			0.152
Primary School	56 (73.7)	20 (26.3)	
Secondary School	39 (84.8)	7 (15.2)	
**Premorbid**			0.727
Yes	13 (81.3)	3 (18.8)	
No	82 (77.4)	24 (22.6)	

a Chi-square Test.

b Independent T-test.

c Variable was included in the multiple logistic regression analysis.

**Table 3:** Univariable analysis of the ergonomics risk factors and MSD.

Item	Musculoskeletal Disorders (N = 122), n (%)		*P*-value^a^
	Yes	No	
**Teaching in standing continuously (>2 h).**^b^			0.046
Never	21 (63.6)	12 (36.4)	
A few days a week	44 (80.0)	11 (20.0)	
Everyday	30 (88.2)	4 (11.8)	
**Working in sitting continuously (>30 min).**			0.482
Never	9 (69.2)	4 (30.8)	
A few days a week	43 (75.4)	14 (24.6)	
Everyday	43 (82.7)	9 (17.3)	
**Writing on the whiteboard (>2 h/day).**			0.430
Never	21 (70.0)	9 (30.0)	
A few days a week	54 (81.8)	12 (18.2)	
Everyday	20 (76.9)	6 (23.1)	
**Squatting or kneeling position (>2 h/day).**			0.789
Never	49 (76.6)	15 (23.4)	
A few days a week	38 (80.9)	9 (19.1)	
Everyday	8 (72.2)	3 (27.3)	
**Bending position (>2 h/day).**			0.754
Never	38 (77.6)	11 (22.4)	
A few days a week	41 (75.9)	13 (24.1)	
Everyday	16 (84.2)	3 (15.8)	
**Carrying teaching materials (> 5 kg).**			0.972
Never	25 (78.1)	7 (21.9)	
A few days a week	44 (78.6)	12 (21.4)	
Everyday	26 (76.5)	8 (23.5)	
**Climbing up or down stairs (>30 stairs).**			0.554
Never	25 (71.4)	10 (28.6)	
A few days a week	41 (80.4)	10 (19.6)	
Everyday	29 (80.6)	7 (19.4)	
**Assist the student in toileting/changing diapers.**			0.929
Never	49 (76.6)	15 (23.4)	
A few days a week	39 (79.6)	10 (20.4)	
Everyday	7 (77.8)	2 (22.2)	
**Assist the student in feeding.**			0.932
Never	46 (76.7)	14 (23.3)	
A few days a week	39 (79.6)	10 (20.4)	
Everyday	10 (76.9)	3 (23.1)	
**Assist the student in ambulation.**			0.851
Never	50 (78.1)	14 (21.9)	
A few days a week	31 (75.6)	10 (24.4)	
Everyday	14 (82.4)	3 (17.6)	

a Chi-square Test.

b Variable was included in the multiple logistic regression analysis.

**Table 4:** Univariable analysis of the psychological factors and MSD.

Item	Musculoskeletal Disorders (N = 122), Mean (SD)		*P*-value^a^
	Yes	No	
**Perceived Stress Scale**^b^	15.99 (4.63)	13.56 (4.50)	0.017
**Mindful Attention Awareness Scale**^b^	4.50 (0.73)	4.82 (0.65)	0.040

a Independent T-test.

b Variable was included in the multiple logistic regression analysis.

**Table 5:** Univariable analysis of the musculoskeletal fitness factors and MSD.

Item	Musculoskeletal Disorders (N = 122), n (%)		*P*-value^a^
	Yes	No	
**Body Fat Percentage**^c^**,** Mean (SD)	32.48 (8.85)	26.98 (8.91)	0.005^b^
**Back Extensor**^c^			0.017
Good (≥101 s)	29 (65.9)	15 (34.1)	
Poor (<101 s)	66 (84.6)	12 (15.4)	
**Left Side Bridge**^c^			0.040
Good (≥42 s)	23 (65.7)	12 (34.3)	
Poor (<42 s)	72 (82.8)	15 (17.2)	
**Right Side Bridge**^c^			0.031
Good (≥42 s)	28 (66.7)	14 (33.3)	
Poor (<42 s)	67 (83.8)	13 (16.3)	
**Plank on Elbow**^c^			0.041
Good (≥83 s)	29 (67.4)	14 (32.6)	
Poor (<83 s)	66 (83.5)	13 (16.5)	
**Left Apley Scratch**			0.569
Good	54 (76.1)	17 (23.9)	
Poor	41 (80.4)	10 (19.6)	
**Right Apley Scratch**^c^			0.030
Good	69 (73.4)	25 (26.6)	
Poor	26 (92.9)	2 (7.1)	
**Chair Rise 30 s**			0.746
Good (≥23 rep)	32 (76.2)	10 (23.8)	
Poor (<23 rep)	63 (78.8)	17 (21.3)	
**Left single-leg squat**			0.896
Good	75 (78.1)	21 (21.9)	
Poor	20 (76.9)	6 (23.1)	
**Right Single Leg Squat**			0.941
Good	78 (78.0)	22 (22.0)	
Poor	17 (77.3)	5 (22.7)	
**Left Forward Lunge**			0.452
Good	83 (76.9)	25 (23.1)	
Poor	12 (85.7)	2 (14.3)	
**Right Forward Lunge**			0.801
Good	86 (78.2)	24 (21.8)	
Poor	9 (75.0)	3 (25.0)	

a Chi-square Test.

b Independent T-test.

c Variable was included in the multiple logistic regression analysis.

## 4 The predictors of MSD

A multiple logistic regression analysis was conducted to identify the predictors of MSD among special education teachers. All the variables from the univariable analysis with a significant level of less than 0.25 were selected for the multiple logistic regression analysis. The multiple logistic regression analysis used Forward Likelihood Ratio (LR) and Backward Likelihood Ratio (LR) methods (Table 6). For both Forward and Backward LR, five variables were retained in the preliminary main effects model: marital status, continuously teaching in a standing position for more than 2 h, body fat percentage, back extensor, and mindfulness, with all variables having significant *P*-value.

**Table 6:** Predictors of musculoskeletal disorders among special education teachers.

Item	Adjusted B *	Adjusted Odds Ratio	95 % Confidence Interval	*P*-value
**Marital Status** Unmarried Married	1.565	1 4.78	1.49, 15.40	0.009
**Body Fat Percentage**	0.060	1.06	1.00, 1.12	0.043
**Teaching in standing continuously (>2 h)** Never A few days a week Everyday	1.163 1.825	1 3.20 6.20	0.99, 10.29 1.44, 26.70	0.034 0.051 0.014
**Mindfulness**	−0.764	0.47	0.22, 0.98	0.044
**Back Extensor** Good Poor	1.769	1 5.86	1.92, 17.92	0.002

Constant = 0.000.

No multicollinearity problem and no interaction.

Hosmer Lemeshow test (p = 0.387).

Sensitivity: 93.7 %, Specificity: 40.7 %, PPV: 84.7 %, NPV: 64.7 %, F1 Score: 0.889.

Classification table: 82.0 %.

AUC: 0.805 (95 % CI = 0.71, 0.90) (p < 0.05).

Cook's influential statistic (data<1.0).

The preliminary model was tested for interaction, and no interaction was found. A collinearity diagnostics test revealed no multicollinearity problem. For overall model fitness, the Hosmer-Lemeshow test confirmed that the model fits well (p = 0.387). Sensitivity, measuring the proportion of actual positive cases correctly identified by the model, was 93.7 %. Specificity, measuring the proportion of actual negative cases correctly identified by the model, was 40.7 %. The Positive Predictive Value (PPV), indicating the proportion of predicted positive cases that are actually positive, was 84.7 %. The Negative Predictive Value (NPV), indicating the proportion of predicted negative cases that are actually negative, was 64.7 %. The model's F1 Score of 0.889 indicated good precision and recall. The accuracy of the classification table in predicting MSD among Special Education Teachers was 82.0 %, indicating that the model correctly classified MSD and non-MSD cases 82.0 % of the time. The Area Under the Receiver Operating Characteristic (ROC) Curve (AUC) was 0.805 (p < 0.05); therefore, the model can accurately discriminate 80.5 % of the MSD cases. Cook's influential statistic was used to check for outliers, and none of our data points had a value greater than 1.0, indicating no influential outliers (Table 6).

Therefore, with a constant of 0.000, the marital status (aOR = 4.78, 95 % CI = 1.49, 15.40, p = 0.009), teaching in a standing position continuously (>2 h) for few days a week (aOR = 3.20, 95 % CI = 0.99, 10.29, p = 0.051) and every day (aOR = 6.20, 95 % CI = 1.44, 26.70, p = 0.014), body fat percentage (aOR = 1.06, 95 % CI = 1.00, 1.12, p = 0.043), back extensor (aOR = 5.86, 95 % CI = 1.92, 17.92, p = 0.002), and mindfulness (aOR = 0.47, 95 % CI = 0.22, 0.98, p = 0.044) were found to be the predictors of MSD among the special education teachers (Table 6).

## 5 Discussion

### 5.1 The prevalence of MSD

MSD among teachers, especially special education teachers, is a significant concern that has been extensively studied worldwide [[27], [28], [29]]. The current study revealed a high prevalence of MSD among special education teachers, with a rate of 77.9 %, which is consistent with rates reported in Kelantan (72 %), Taiwan (86 %), and Italy (85.9 %) [2,8,30]. One plausible explanation for the disparities in MSD prevalence among teachers across countries and regions is the variation in workloads and work environments. Special education teachers often face additional physical tasks, such as lifting and transferring students, which can contribute to a higher prevalence of MSD compared to regular teachers [[31], [32], [33]]. Furthermore, differences in the availability of ergonomic equipment and training in various countries may also influence the prevalence of MSD among teachers [30,34,35].

Analyzing the specific body regions affected by MSD in this study, special education teachers exhibited the highest prevalence of MSD in the lower back (60.7 %), followed by the shoulder (56.6 %) and neck (53.3 %). These findings align with previous studies conducted among special education teachers in Taiwan and Kelantan, which reported similar patterns of affected body regions [1,2,8]. This study has demonstrated that poor fitness, encompassing strength and endurance in the back, body core, and lateral core areas, significantly contributes to the high prevalence of MSD in the lower back region among special education teachers. Additionally, ergonomic risks associated with job tasks that require teachers to stand for prolonged periods exceeding 2 h also contribute significantly to MSD in the back region within this study. Moreover, the study has indicated that poor shoulder flexibility among special education teachers is believed to contribute to the high prevalence of MSD in the shoulder and neck regions due to muscular imbalances, increased muscle tension, and impaired circulation and nerve function.

According to previous studies, activities such as assisted toileting and rehabilitation were identified as potential risk factors for MSD, particularly in the lower back region, as teachers frequently bend their waist and knees to accommodate the height of children or the size of furniture [1,36]. A study conducted in Germany also highlighted the significantly higher prevalence of chronic back pain among special education teachers involved in nursing care tasks, such as carrying, lifting, transferring pupils, washing pupils, providing toilet assistance, changing diapers, and helping with dressing [7].

Cultural attitudes toward work-related injuries and illnesses may contribute to the differences in MSD prevalence among teachers as well. In some cultures, there may be a greater stigma associated with reporting work-related injuries, leading to the potential underreporting of MSD cases among teachers [37]. Despite the underlying reasons for the variations in MSD prevalence, it is evident that MSD represents a significant issue that necessitates attention and action. Extensive literature reviews have highlighted the substantial physical and emotional consequences of MSD on teachers, impeding their ability to perform their jobs effectively [38,39].

### 5.2 Associated factors of MSD

The factors associated with MSD among special education teachers are multifaceted, encompassing both personal and occupational aspects. Marital status and BMI are among the personal factors associated with MSD prevalence in this group. Married teachers exhibited a higher incidence of MSD, potentially due to additional home responsibilities [40]. In contrast, unmarried teachers in a German study reported higher rates of chronic back pain, possibly related to social isolation and increased stress levels [7]. However, most studies found no significant correlation between marital status and MSD [[41], [42], [43]]. A high BMI is significantly correlated with MSD, as overweight and obese individuals are more prone to lower back and knee pain [[44], [45], [46]]. Excessive body weight can lead to poor posture, increased muscle and joint strain, and a higher MSD risk [47].

Though not statistically significant, factors such as older age, female gender, higher education levels, non-smoking, longer teaching experience, secondary school teaching, and premorbid conditions were associated with a higher MSD incidence, aligning with prior research [2,37]. Age-related physiological changes, prolonged exposure to demanding teaching conditions, and pre-existing medical conditions increase MSD risk [48]. Additionally, while not significant in this study, smoking has been linked to MSD due to impaired tissue repair and decreased bone density [49,50]. The intricate nature of MSD and the interaction between sociodemographic and occupational risk factors highlight the need to consider both individual and occupational factors in developing MSD prevention and management interventions for special education teachers [51,52].

Ergonomic risk factors significantly influence MSD prevalence among special education teachers. This study found that standing continuously for more than 2 h daily and several days per week was associated with a higher MSD incidence compared to teachers who did not stand as long. This finding particularly pertains to the increased MSD incidence in the lower back, consistent with previous studies that identified static and sustained postures as significant ergonomic risk factors for MSD among special education teachers [33,53]. Prolonged standing increases pressure on joints and muscles, causing discomfort and pain in areas such as feet, ankles, knees, hips, and lower back. Extended standing can lead to muscle fatigue, reduced shock absorption, impaired leg and foot blood flow, and increased swelling and discomfort [54,55].

Conversely, this study found no significant differences in other ergonomic risk factors, such as continuous sitting or writing on different whiteboard parts. These results contrast with research from Ethiopia, Australia, and Thailand, which identified associations between specific static postures and MSD among teachers [27,43,56]. Likewise, no significant correlations were found between various physical tasks performed by special education teachers, like squatting, kneeling, bending, carrying heavy materials, and assisting students in daily activities, and the occurrence of MSD. However, studies from Germany and Japan reported different findings regarding heavy lifting and assisting with mobility and daily functions [7,29].

Perceived stress is a significant MSD contributor among special education teachers, supporting previous study findings [38,57]. The link between stress and MSD can be attributed to factors like stress-induced inflammation, physiological responses such as increased muscle tension and pain sensitivity, and stress-related changes in posture, movement, and muscle activation [58,59]. Special education teachers often face high-stress levels due to educational system demands, like excessive workload, large class sizes, extensive paperwork, time constraints, interruptions, and deadlines [60]. For example, a Nigerian study found that a significant number of special education teachers experienced psychological distress, exceeding general population rates [61].

This study explored the relationship between musculoskeletal fitness factors and MSD among special education teachers, finding significant associations. Higher body fat percentage, poor strength and endurance of the back extensor, core trunk, and lateral core, and poor shoulder flexibility and mobility were linked to MSD. These findings are in line with previous research that connected musculoskeletal fitness to MSD occurrence [24,62,63]. Consistently, a higher body fat percentage was associated with MSD among special education teachers, as previous studies indicated that excess body fat intensifies the load on weight-bearing joints and contributes to inflammation related to musculoskeletal pain [64,65].

The study established that inadequate fitness of the back, core, and lateral core muscles were linked to an increased incidence of MSD in the lower back region, while poor shoulder flexibility was associated with a higher prevalence of MSD in the shoulder and neck areas among special education teachers. Weak back and abdominal muscles can amplify stress on the lower back, heightening the risk of lower back pain [56,66]. Similarly, insufficient strength and endurance in the body core and lateral core muscles, which stabilize the spine, were connected to higher MSD occurrences. Core stability is vital for preventing excessive spinal movements and reducing MSD risk, underscoring the importance of core muscle training and strengthening in preventing MSD [67,68]. Weak core muscles can result in compensatory movements and overuse injuries in other body parts [67,69]. Moreover, limited shoulder flexibility and mobility were linked to higher MSD occurrences, as restricted shoulder movement can lead to shoulder impingement syndrome, a common MSD in occupations involving repetitive overhead activities [24,66]. Therefore, this study emphasizes the significance of addressing musculoskeletal fitness factors in preventing MSD among special education teachers.

### 5.3 The predictors of MSD

The present study identified several predictors of MSD among special education teachers, including marital status, prolonged standing for over 2 h, body fat percentage, back extensor strength, and mindfulness. Married teachers had nearly a fivefold higher risk of MSD compared to unmarried teachers. Standing several days a week increased the likelihood of MSD by more than three times, while daily standing raised the risk by over six times. Each 1 % increase in body fat percentage was associated with 6 % higher odds of MSD. Conversely, individuals with stronger back extensor muscles had almost a sixfold lower likelihood of MSD. Moreover, teachers with higher mindfulness levels exhibited a nearly 50 % reduced risk of MSD.

While specific previous studies on MSD predictors among special education teachers are lacking, a comparison with research on regular teachers reveals relevant insights. A study in Kota Kinabalu identified workplace physical hazards, psychosocial factors, and general well-being as significant predictors of MSD among teachers [32]. Similarly, a study in Selangor highlighted anxiety and lifting heavy weights as predictors of lower back pain [57]. Although our results differ from those of these studies, they reinforce the significant roles of sociodemographic, ergonomic risks, psychological, and musculoskeletal fitness factors in predicting MSD among teachers.

### 5.4 The strengths and limitations

This study is characterized by several notable strengths. Firstly, it achieved a high response rate of 95 %, which enhances the credibility of the data and the reliability of the findings. Secondly, the research employed comprehensive assessment methods, including self-administered questionnaires and musculoskeletal fitness assessments, providing a holistic view of the factors associated with MSD. Thirdly, the study utilized robust statistical analysis techniques, specifically multiple logistic regression, to identify predictors of MSD, offering a nuanced understanding of the contributing factors. Additionally, the study focused on special education teachers, a group often neglected in MSD research, thereby filling a significant gap in the existing literature. The identification of multiple predictors of MSD, such as marital status, body fat percentage, prolonged standing, mindfulness, and back extensor strength, reveals a multifaceted approach to understanding the condition. Lastly, the study's findings have practical implications, emphasizing the importance of considering ergonomic, psychological, and musculoskeletal fitness factors in managing MSD and suggesting that interventions should be comprehensive and targeted to enhance the well-being of special education teachers effectively.

This study has several limitations that are important to consider. It involved a specific group of 122 special education teachers from Kota Kinabalu and Penampang, which may not be representative of all special education teachers, thus limiting the generalizability of the findings to other regions or populations. The cross-sectional design of the study only allows for the identification of associations between variables at a single point in time, which means it cannot establish causal relationships or track the progression of musculoskeletal disorders over time. Additionally, the study relied heavily on self-reported data through questionnaires, which can introduce biases due to the possibility of participants underreporting or overreporting their symptoms or behaviors. The selection process for participants might also have introduced selection bias, potentially skewing the results towards individuals more aware of or affected by musculoskeletal disorders. The lack of longitudinal follow-up prevents the study from examining the long-term effects of identified risk factors on the development or progression of musculoskeletal disorders.

## 6 Conclusions

This study reveals a high prevalence of MSD among special education teachers, particularly affecting the lower back, shoulder, neck, knee, upper back, and foot. Predictors of MSD identified include marital status, body fat percentage, prolonged standing, mindfulness, and back extensor strength. These findings emphasize the importance of considering psychological and musculoskeletal fitness factors alongside sociodemographic and ergonomic factors when addressing MSD among special education teachers. A comprehensive and targeted approach is crucial for effectively managing musculoskeletal health in this population.

The study contributes to our understanding of musculoskeletal fitness and highlights its significance for occupational health and safety. It has practical implications, emphasizing the need for comprehensive strategies in managing MSD among special education teachers. Further research should explore additional predictors of MSD and develop targeted interventions that comprehensively promote musculoskeletal health and prevent MSD. By prioritizing musculoskeletal well-being, we can enhance the overall health and job performance of special education teachers, enabling them to provide quality education and support to children with special needs.

## 7 Recommendations

To effectively address the prevalence of Musculoskeletal Disorders (MSD) among special education teachers, a multifaceted approach is essential. Firstly, ergonomic assessments should be conducted to tailor interventions to the specific needs of the educational environment, identifying and mitigating potential ergonomic risk factors [19]. Customized ergonomic training should then be provided, focusing on proper body mechanics and safe lifting techniques, to educate teachers on minimizing their risk of MSD [70,71].

In addition to ergonomic solutions, the importance of rest and physical activity cannot be overstated. Schools should implement policies that mandate regular breaks and encourage physical activities, thus facilitating recovery from sustained postures and reducing muscle fatigue [34,72]. Access to adjustable furniture and assistive devices should be provided to support proper posture and minimize strain [35].

Psychological well-being is also pivotal in managing MSD risks. Mindfulness-based interventions have proven effective in reducing stress and improving emotional regulation among teachers, which can, in turn, alleviate muscle tension and contribute to better musculoskeletal health [[73], [74], [75], [76]]. Techniques such as deep breathing, body scanning, and mindful movement should be integrated into teacher wellness programs to promote relaxation and improve body awareness, thus reducing the likelihood of MSD occurrence [77,78].

Finally, targeted exercises and wellness programs designed to enhance musculoskeletal fitness should be implemented. These programs should aim not only at reducing the immediate risk of MSD but also at promoting long-term health and well-being among special education teachers. Through a comprehensive and proactive approach encompassing ergonomic improvements, physical activity, psychological support, and targeted wellness initiatives, schools can create a healthier and more supportive work environment for teachers, thereby reducing the incidence and impact of MSD.

## FUNDING

This research received funding from the Fundamental Research Grant Scheme (FRGS), 10.13039/501100002385Ministry of Higher Education Malaysia (Code project no.: FRGS/1/2022/SKK04 /UMS/02/1).

## Ethical statement

The study was conducted in accordance with the Declaration of Helsinki and approved by the Medical Research Ethics Committee, Faculty of Medicine and Health Science, Universiti Malaysia Sabah (Approval code: JKEtika 2/21(14)) on August 17, 2021.

## Informed consent statement

Informed consent was obtained from all subjects involved in the study.

## Data availability statement

• Data associated with the study has NOT been deposited into a publicly available repository.
• Data will be made available on request.

## CRediT authorship contribution statement

**Mohammad Saffree Jeffree:** Writing – review & editing, Data curation, Conceptualization. **Ahmad Asyraf Abdul Rahim:** Writing – original draft, Formal analysis, Data curation, Conceptualizatio. **Dayang Maryama Ag Daud:** Writing – review & editing, Conceptualization. **Nicholas Pang:** Writing – review & editing, Data curation, Conceptualization. **Mohd Fazeli Sazali:** Writing – review & editing, Writing – original draft, Conceptualization. **Suhaini Sudi:** Writing – review & editing, Conceptualization. **Shye Nee Liau:** Writing – original draft, Conceptualiza. **Eileen Ei Ling Wong:** Writing – original draft, Conceptualization. **Hanif Fikri Peter:** Writing – original draft, Conceptualization. **Siti Zuraina Ain Amat:** Writing – review & editing, Conceptualization. **Stephanie Chok:** Writing – original draft, Conceptualization. **Mohsen M.A. Abdelhafez:** Writing – original draft, Conceptualization. **Khamisah Awang Lukman:** Writing – original draft, Formal analysis, Conceptualization. **Ismail Saad:** Writing – original draft, Conceptualization. **Mohd Rohaizat Hassan:** Writing – review & editing, Conceptualization. **Raman Noordin:** Writing – original draft, Conceptualization.

## Declaration of competing interest

The authors declare the following financial interests/personal relationships which may be considered as potential competing interests:Mohammad Saffree Jeffree reports financial support wras provided by Fundamental Research Grant Scheme (FRGS). If there are other authors, they declare that they have no known competing financial interests or personal relationships that could have appeared to influence the work reported in this paper.
